# A Comparison of Ovine-Reinforced Hybrid Mesh (OviTex PRS) With Porcine Acellular Dermal Matrix (STRATTICE) in the Treatment of Advanced Breast Implant Capsular Contracture

**DOI:** 10.1093/asjof/ojae068

**Published:** 2024-08-20

**Authors:** Cyril J Harfouche, Michael J Brucker, Salvatore J Pacella

## Abstract

**Background:**

Tissue reinforcement techniques with porcine acellular dermal matrices (ADMs) have been widely adopted as standard care in treating capsular contracture. However, the application of alternative xenograft or mesh materials has not been widely studied.

**Objectives:**

To examine the efficacy of OviTex PRS Reinforced Tissue Matrix (Resorbable) (TELA Bio, Malvern, PA), a hybrid ovine-reinforced mesh, in comparison with STRATTICE Reconstructive Tissue Matrix (Allergan, Irvine, CA), in patients with advanced capsular contracture.

**Methods:**

A retrospective review was conducted on patients who underwent breast revision surgery for Baker Grade III or IV capsular contracture. Patient data were reviewed for outcomes, complications, cost, and postoperative incidence of recurrent capsular contracture after treatment with each specific mesh.

**Results:**

Fifty-nine of 89 breasts (66.3%) were treated with OviTex and 30 (33.7%) were treated with STRATTICE. All patients experienced a reduction in Baker grades. In patients treated with OviTex, 96.6% (*n* = 57) of breasts had a postoperative Baker Grade I with the remaining 3.4% (*n* = 2) breasts presenting with a Baker Grade II. In comparison, 73.3% (*n* = 22), 23.3% (*n* = 7), and 3.3% (*n* = 1) of the STRATTICE cohort presented with Baker Grades I, II, and III, respectively. This demonstrated a statistically significant improvement in Baker grade capsular contracture in the OviTex cohort (*P* < .05) compared with STRATTICE. Average cost was $27.37/cm^2^ for STRATTICE compared with $22.28/cm^2^ for OviTex PRS.

**Conclusions:**

OviTex may be successfully utilized to treat capsular contracture. Patient outcomes may be superior to STRATTICE in recurrent capsular contracture, particularly when a previous ADM had been utilized. Cost data show improved savings with the use of OviTex compared with STRATTICE.

**Level of Evidence: 3:**

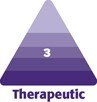

Despite advancements in breast implant surface technology, capsular contracture remains the most common and insidious complication in modern cosmetic breast augmentation.^1^ With incidence rates varying from 2% to 15% following primary breast augmentation, it is one of the leading indications for revisional augmentation procedures in the United States.^2^

On a cellular level, capsular contracture is a pathologic and inflammatory tissue response arising from the interface of implanted material to the native fibrous capsule in breast tissue, which commonly presents as pain, tenderness, firmness, and cosmetic deformity. It is commonly accepted that the etiology of capsular contracture arises from the development of chronic inflammation caused by subclinical infection with subsequent biofilm formation.^3-8^ Surgical techniques, such as the rinsing of implants with betadine or antibiotic mixtures, have evolved to mitigate the risk of development of biofilm.^9^ In addition, strategies for reduction of capsular contracture, such as placement of textured implants, submuscular placement, avoidance of subclinical hematoma formation, and prophylactic treatment with leukotriene receptor agonists, are also widely accepted.^10-13^ Despite the wide adoption of these techniques, the risk of development is still common.^1^

In the provider community, capsulectomy (with or without pocket revision) and implant exchange is commonly considered to be the gold standard of surgical management for capsular contracture.^14^ Nonetheless, clinical evidence to support this as a reproducible therapy is insufficient. This, in turn, has led to the use of alternative strategies for treatment in revisional breast surgery.

Pocket revision with acellular dermal matrices (ADMs) has become widely adopted as effective treatment for recurrent capsular contracture.^15^ The earliest studies have demonstrated efficacy with the use of human ADMs; however, the cost and availability of these products have presented economic and logistical challenges.^15-18^ The use of porcine-based and/or xenograft ADMs has been extrapolated for use as an alternative treatment, despite limited clinical evidence of its efficacy.^15-22^

Ovine (ie, sheep rumen/foregut) reinforced hybrid mesh has been previously shown to be safe and effective in abdominal wall reconstruction and hernia surgery, demonstrating patient satisfaction and exceptionally low recurrence rates.^23^ The results of human clinical and nonhuman primate studies have previously demonstrated that ovine-reinforced hybrid mesh had a limited foreign body response, earlier cellular infiltration and facilitated remodeling response.^24^ It is hypothesized that this hybrid mesh may combine the benefits a biologic matrix (ie, lower inflammatory profile, incorporation, vascularity, compliance) with the long-term structural integrity of a synthetic polymer.^25^

To date, there have been no studies documenting the efficacy of ovine-based or hybrid biologic/synthetic meshes in the treatment of capsular contracture. In addition, this type of mesh has never been evaluated in comparison with standard porcine matrices that are commonly utilized for revisional aesthetic breast surgery. In this manuscript, we present a consecutive clinical comparison of OviTex PRS Reinforced Tissue Matrix (Resorbable; TELA Bio, Malvern, PA) to a 2-ply reinforced resorbable matrix to porcine-derived mesh (ie, STRATTICE; Allergan, Irvine, CA) in the treatment of capsular contracture.

## METHODS

### Patient Selection

A retrospective analysis was conducted by 2 primary surgeons from May 2016 to November 2023. Patients undergoing revisional breast augmentation for Baker Grade III or IV capsular contracture utilizing STRATTICE or OviTex PRS Resorbable were included in the study. This study was designed in accordance with the guidelines of the Department of Health and Human Services Regulations for the Protection of Human Subjects, Belmont Report and Declaration of Helsinki. This study used retrospective, de-identified patient information who provided written informed consent preoperatively. Institutional review board approval was obtained for this study. Patient records were reviewed for age, surgical history, previous implant location, before ADM use, complications or reoperations, length of follow-up, and any secondary procedures performed. Inclusion criteria included patients >18 years old with a history of breast augmentation surgery and subsequent Baker Grade III or IV capsular contracture that was treated utilizing OviTex PRS or STRATTICE mesh. Exclusion criteria included inadequate follow-up (<3 months), revision without the use of mesh, and history of breast reconstruction after breast cancer or radiation exposure. Patient demographics are illustrated in Table 1.

**Table 1.** Patient Demographic and Outcome Variables in Capsular Contracture Treatment with OviTex PRS vs STRATTICE

Demographics	Total (%)	OviTex PRS	STRATTICE	*P*-value
No. of patients	50	33 (66%)	17 (34%)	
No. of breasts	89	59 (66.3%)	30 (33.7%)	
Age at the time of surgery	55.9 ± 11.9	56.8 ± 12.6	54.3 ± 10.6	.373
No. of previous revisions	1.8 ± 1.1	1.8 ± 1.3	1.9 ± 0.7	.305
Follow-up duration	357 ± 206	308 ± 279	453.8 ± 585	.296
Capsulectomy type^a^				.503
Total	55 (61.7%)	35 (59.3%)	20 (66.7%)	
Subtotal	34 (38.2%)	24 (40.7%)	10 (33.3%)	
Concurrent mastopexy^a^				.283
None	51 (57.3%)	32 (54.2%)	19 (63.3%)	
Primary	33 (37.1%)	22 (37.3%)	11 (36.7%)	
Secondary	5 (5.6%)	5 (8.5%)	0	
Time until drain removal, days	6 ± 3.2	5.5 ± 3.1	7.1 ± 3.4	.092
Previous ADM (STRATTICE)^a^	12 (13.5%)	4 (6.7%)	8 (26.7%)	.028
Implant location^a^				.291
Subglandular	24 (26.9%)	18 (30.5%)	6 (20%)	
Subpectoral	65 (73.1%)	41 (69.5%)	24 (80%)	
Implant ruptured^a^	26 (29.2%)	19 (32.2%)	7 (23.3%)	.387
Plane change^a^				.336
None	66 (74.2%)	42 (71.2%)	24 (80%)	
Subpectoral to subglandular	4 (4.5%)	2 (3.4%)	2 (6.7%)	
Subglandular to subpectoral	19 (21.3%)	15 (25.4%)	4 (13.3%)	
Postoperative Baker grade^a,b^				.015
I	79 (88.8%)	57 (96.6%)	22 (73.3%)	
II	9 (10.1%)	2 (3.4%)	7 (23.3%)	
III	1 (1.1%)	0	1 (3.3%)	
IV	0	0	0	

^a^Reporting per breast. ^b^Preoperatively, all breasts in the study had a Baker Grade III or IV capsular contracture.

### Surgical Technique

All patients were treated with implant removal and subtotal or total capsulectomy (when technically feasible). After explant, the breast pocket was irrigated with antibiotic and/or antiseptic solution, followed by implant replacement with or without pocket conversion (ie, maintenance of the subpectoral pocket, subpectoral to subglandular, or subglandular to subpectoral). In subpectoral implant replacement, mesh was fixated to the free lower border of the pectoralis muscle. In subglandular replacement, mesh was either fixated to the upper border of the pectoralis (if the vertical dimension of the mesh allowed fixation without significant tension) or fixated freely to the breast parenchyma utilizing 2-0 polydiaxanone, leaving the most superior aspect of the implant uncovered.

Laterally and medially, a wrap-around fixation technique was utilized to partially cover the posterior aspect of the implant in a 1 to 2 cm underlay. All implants were standard (MENTOR MemoryGel Smooth Round, various profiles, Santa Barbara, CA) and were inserted utilizing a no-touch technique (Keller funnel) rinsed with dilute betadine. In the lower pole, the mesh was placed as an onlay posterior to the implant to create an inferiorly based sling and tangentially sutured to the most inferior mesh border after the implant was inserted. OviTex PRS mesh placement and fixation are illustrated in Video.

In the STRATTICE cohort, a 9 × 18.5 cm (133 cm^2^) piece of mesh (STRATTICE Contour 2) was utilized. An 8 × 15 cm (99 cm^2^) piece was utilized in the OviTex PRS cohort. Drains were utilized in each case and standardly removed when output reduced to below 30 cc/day.

Patient outcome variables included the presence of any perioperative or postoperative complication, reoperations, days until drain removal, the presence of infection or seroma, as well as any recurrence in capsular contracture throughout their follow-up period.

### Statistical Analysis

Statistics were performed utilizing IBM SPSS v28.0 (Armonk, NY). Statistical methods employed include descriptive statistics and Wilcoxon signed-rank sum test to compare the primary outcome measure between both groups as well as the other clinical and demographic variables.

### Cost

Cost of each type of mesh was determined and compared per cm^2^ based on standard pricing from each vendor.

## RESULTS

A total of 89 breasts (in 50 patients) were included for analysis during the 7-year study period. The mean age at the time of treatment was 55.9 ± 11.9 years. Of the 89 breasts in the consecutive study, 30 breasts were treated with STRATTICE and 59 were treated with OviTex PRS. The mean length of follow-up after surgery was 357 ± 206 days. There were no statistically significant differences between both groups in the mean age of patients, follow-up duration, number of previous revisional surgeries, implant location, concurrent mastopexy, concurrent capsulectomy, and plane change (*P* > .05). Breasts in the STRATTICE cohort were more likely to have had before ADM than the OviTex PRS cohort, with rates of 26.7% (*n* = 8) and 6.7% (*n* = 4), respectively (*P* = .028). After treatment, patients treated with OviTex PRS demonstrated a statistically significant reduction in Baker grade capsular contracture compared with patients treated with STRATTICE using a Wilcoxon rank-sum test (*P* = .015).

During the follow-up period, 1 patient in the OviTex PRS developed a hematoma on postoperative Day 1 which required surgical evacuation. There were no additional complications (ie, infection, seroma, hematoma, or explantation) in either cohort. There were no statistical differences with regard to length of days required for drain removal (OviTex PRS: 5.5 ± 3.1 days, STRATTICE: 7.1 ± 3.4 days; *P* = .092).

**Figure 1.**
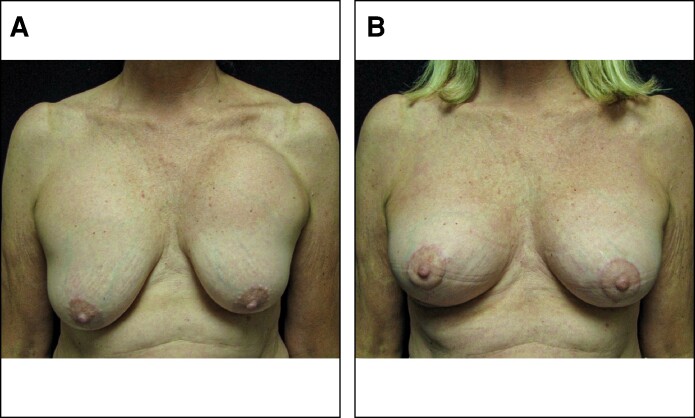
Treatment of advanced bilateral capsular contracture with OviTex PRS (TELA Bio). (A) A 58-year-old female presenting with Baker Grades III and IV capsular contracture in the left and right breast, respectively. (B) Postoperative results are demonstrated 12 months following capsulectomy, circumareolar mastopexy, implant exchange, and OviTex PRS placement.

**Figure 2.**
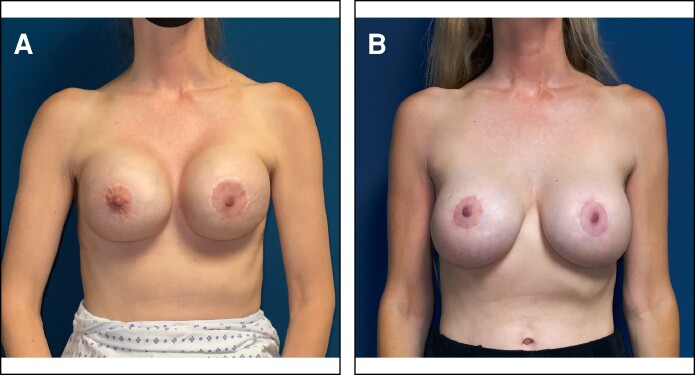
Treatment of advanced bilateral capsular contracture with OviTex PRS (TELA Bio). (A) A 50-year-old female presenting with bilateral Baker Grade IV capsular contracture desiring revision. (B) Postoperative results at 13 months following capsulectomy, implant exchange, circumareolar mastopexy, and OviTex PRS placement.

**Figure 3.**
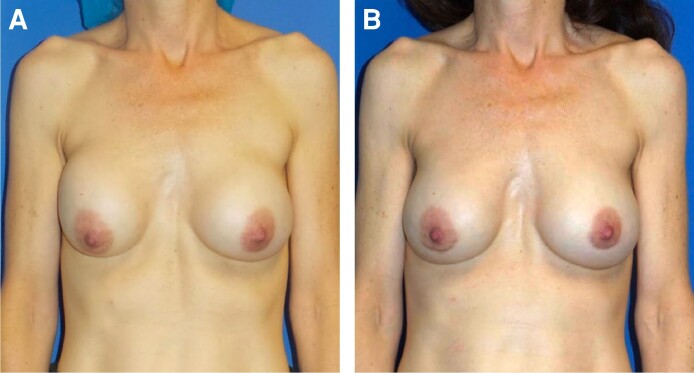
Treatment of advanced bilateral capsular contracture with STRATTICE (Allergan). (A) A 42-year-old female presenting with bilateral Baker Grade IV capsular contracture. (B) Postoperative results are demonstrated at 12 months following capsulectomy, implant exchange and Strattice placement.

In patients treated with OviTex PRS, 96.6% (*n* = 57) of breasts had a postoperative Baker Grade I with the remaining 3.4% (*n* = 2) breasts presenting with a Baker Grade II. In comparison, 73.3% (*n* = 22), 23.3% (*n* = 7), and 3.3% (*n* = 1) of the STRATTICE cohort presented with Baker Grades I, II, and III, respectively (Table 1). Sample patient results from each cohort are illustrated in Figures 1 to 3.

**Figure 4.**
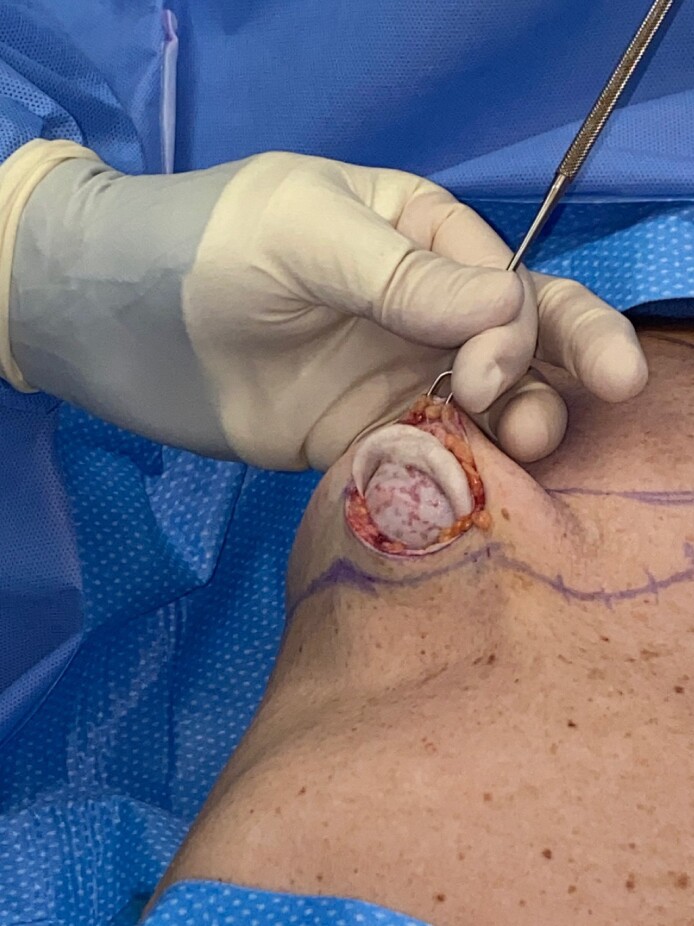
Recurrence of Baker Grade IV capsular contracture following treatment with STRATTICE (Allergan). Intraoperative view of recurrent Baker Grade IV capsular contracture in a 49-year-old female patient with existing STRATTICE placed 2 years before presentation. Visualization of the breast pocket demonstrates incorporation of the biologic mesh integrated with fibrous tissue formation and calcification. The patient required complete capsulectomy, implant exchange, and mesh replacement.

In a small subcohort, 12 breasts (*n* = 6 patients) presented with a recurrent Baker Grade III/IV capsular contracture despite the previous use of STRATTICE. In this group, 8 breasts (*n* = 4 patients) were subsequently treated with revisional capsulectomy and STRATTICE placement, while 4 breasts (*n* = 2 patients) were treated with OviTex PRS. No statistical conclusion could be determined regarding the change in Baker grade between both groups postoperatively due to the small sample size. An intraoperative photograph of recurrent capsular contracture after STRATTICE placement is illustrated in Figure 4.

**Figure 5.**
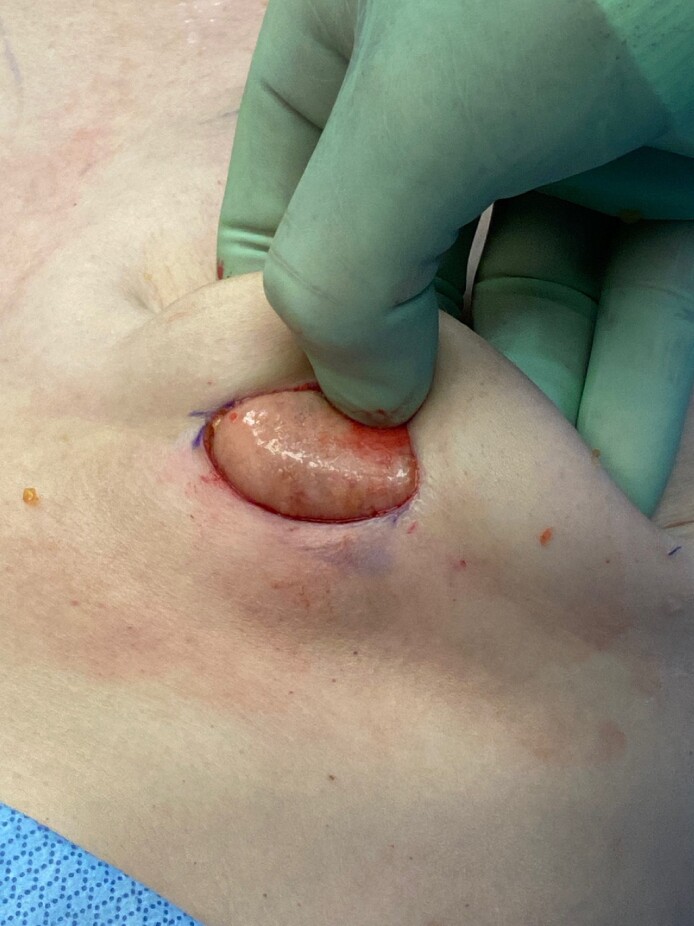
Incorporation of OviTex PRS Resorbable (TELA Bio) after treatment of recurrent capsular contracture followed by implant size change. A 50-year-old female who was successfully treated for recurrent capsular contracture with capsulectomy, OviTex PRS placement, and implant replacement. At 12 months, the patient requested an implant exchange to increase her breast size. Visualization of the breast pocket during this exchange surgery demonstrates vascularized incorporation of the device with thin capsule formation and pliability.

One patient in the OviTex PRS cohort requested reparation to increase implant size, which allowed visualization of the incorporated mesh (Figure 5). There were no other reoperations in either cohort.

Based on a per cm^2^ analysis, STRATTICE BPS Contour 2 was found to be more expensive ($27.37/cm^2^) compared with OviTex PRS ($22.28/cm^2^).

## DISCUSSION

Despite a broader scientific understanding of the etiology of capsular contracture in revisional aesthetic breast surgery, optimal clinical management remains elusive.^26^ Several early generation studies have demonstrated some clinical efficacy in the use of human ADMs (ie, Alloderm, Flex HD) in revisional aesthetic breast surgery; however, these are not consistent or conclusive.^15,27,28^ Although most studies evaluating human ADM use for capsular contracture in breast cancer reconstruction show some efficacy, aesthetic surgeons often anecdotally avoid their use due to ethical concerns in cosmetic-based patients. In addition, the exceptional cost of human ADMs renders them prohibitive for many cosmetic patients.^29^

As a consequence, xenograft biologic matrices have been extrapolated for use in the treatment of capsular contracture. Most commonly, the porcine ADM STRATTICE has demonstrated modest clinical efficacy, with low complication rates.^15,18,20,21,30^ In a recent systematic review and meta-analysis, Samuels et al reported varying success between different human and nonhuman products used across studies, with STRATTICE found to be more efficacious in reducing capsular contracture when compared with other products.^31^ The extrapolation of these xenograft products to aesthetic breast revision can be attributed to their similar industry production processes to human ADMs as well as their performance in abdominal wall reconstruction.^32,33^

OviTex 1S, a 6-ply ovine-reinforced hybrid mesh, has recently demonstrated clinical efficacy in abdominal wall reconstruction with exceptionally low recurrence rates.^23,34-36^ Its successful use in vivo is thought to be derived from improved functional remodeling and neovascularization as well as integration into host tissue with a limited foreign body response when compared with other mesh products compared in preclinical studies.^11,22-24^ Although this is clinically demonstrated in the breast in Figure 5, the authors have also anecdotally witnessed the facility of incorporation of the product in breast reconstruction during implant exchange from tissue expander. We feel that its incorporation in the use in aesthetic breast surgery (where the perfusion of the pocket is much more vascular) is clearly adaptable.

In this study, we have utilized a 2-ply construct of a hybrid ovine-reinforced resorbable mesh (OviTex PRS), which has been specifically re-designed for use outside of the abdominal wall (and potentially in the breast). Although having FDA 510(k) clearance for soft-tissue reinforcement, to date, there have been no studies documenting its efficacy specifically for use in revisional aesthetic breast surgery.

In this consecutive study, 95% of the study group presented with recurrence of capsular contracture in the setting of previous surgical treatment with capsulectomy or porcine ADM (ie, STRATTICE) placement. In the author's experience, before the initiation of this study, there was anecdotal observation that a small cohort of patients had advanced recurrence of Baker Grade III/IV capsular contracture despite original treatment with STRATTICE. This is consistent with the observed cohort which was statistically found to be more likely to have had previous ADM placed than the OviTex PRS cohort (Table 1, previous ADMs). The observation of this subset demonstrates the need for potential alternative treatments after a porcine ADM has failed (Figure 4). These data also support the use of an alternative and less expensive mesh in recurrent capsular contracture after failed initial treatment with a porcine ADM.

To date, no studies have examined the use of OviTex PRS Resorbable in breast reconstructive or aesthetic surgery. In comparing the incidence of postoperative capsular contracture, OviTex PRS demonstrated a statistically significant improvement in Baker grade when compared with STRATTICE. This difference was also statistically significant when accounting for confounding variables. Despite these results, this study has several limitations.

Studies have previously demonstrated that capsular contracture can develop as early as 1 to 2 months to as many as several years after breast augmentation.^1^ With an average follow-up of 11.3 months (ie, 357 days), it is certainly possible that patients in this retrospective study may go on to develop capsular contracture in the future, thereby underestimating the long-term incidence. In this study, our endpoints may only conclude improvement in capsular contracture within the first year (on average) as well as maximum follow-up time (2.8 years for STRATTICE, 1.6 years for OviTex PRS). Further investigation into the long-term benefits of OviTex PRS placement is clearly warranted. In the future, prospective, randomized, controlled, and multi-year studies would be beneficial to specifically determine which mesh and/or biologic, if any, is effective in preventing the lifetime risk of recurrent capsular contracture.

In addition, while the study was consecutive, it was not randomized. In the authors' practices, STRATTICE was available in the marketplace before OviTex PRS. A majority of the patients undergoing STRATTICE placement were clearly in the earlier years of the study, which can certainly introduce a time-based selection bias with regard to surgeon's choice of mesh. Furthermore, the subcohort of recurrent capsular contracture after initial treatment of STRATTICE, may introduce selection bias to choose an alternative product when the initial treatment has failed.

Finally, Baker grade, while classically utilized as a clinical grading tool for capsular contracture, may be unreliable as a grading system for performing scientific research. A recent study concluded that interobserver reliability of the Baker classification system is poor and may be subject to different interpretations by different surgeons.^37^ Further investigations may develop a more reliable scale and/or utilize histopathologic grading systems which may be more beneficial to conclude scientifically.^38^

An additional factor to be considered during preoperative planning is the cost associated with the use of xenograft products, which is often passed on to the patient as consumer. In this study, based on general cost in the marketplace, OviTex PRS Resorbable clearly demonstrates significant cost savings of $5.09/cm^2^ when compared with STRATTICE. While both products performed similarly with minimal complications and no differences in potential need for extended drain placement, and demonstrate similar efficacy, substantial cost savings may influence potential choice of mesh type. This, in turn, may ease the financial burden for patients who may have repeatedly necessitated paying for the continued cost of treating the complication of their revisional breast surgery.

## CONCLUSIONS

With the wide variety of xenografts marketed to treat capsular contracture, optimal choice should be based on clinical superiority balanced with cost efficacy. Our data suggest that OviTex PRS Resorbable is an equivalent and statistically superior, safe, and cost-effective option for patients with Baker Grade III/IV capsular contracture, when compared with STRATTICE. In addition, it may demonstrate efficacy in a subset of patients who failed previous treatment with porcine-derived ADMs.
